# Cyclopalladated Complexes With Functionalized Diphosphanes as Promising Antifungal Scaffolds

**DOI:** 10.1155/bca/6220526

**Published:** 2026-04-11

**Authors:** Cláudia Malta-Luís, Carolina Mariano, Teresa Monteiro, Francisco C. Mendes, María Villar-López, Álvaro J. Arana, Laura Sánchez, Digna Vázquez García, Alberto Fernández, José M. Vila, Jesús J. Fernández, Oscar Lenis-Rojas, Catarina Pimentel

**Affiliations:** ^1^ Instituto de Tecnologia Química e Biológica António Xavier, Universidade Nova de Lisboa, Av. República Oeiras, Lisboa, 2780-157, Portugal, unl.pt; ^2^ Departamento de Zoología, Genética y Antropologia Física, Facultad de Veterinaria, Universidad de Santiago de Compostela, ES 27002 Santiago de Compostela, Coruña, Spain, usc.es; ^3^ Área de Química Inorgánica, Departamento de Química, Facultad de Ciencias, Universidade da Coruña, Coruña, A Coruña, 15008, Spain, udc.es; ^4^ Centro de Investigaciones Científicas Avanzadas (CICA), Universidade da Coruña, Coruña, A Coruña, 15008, Spain, udc.es; ^5^ Departamento de Química Inorgánica, Facultad de Química, Universidad de Santiago de Compostela, ES 15702 Santiago de Compostela, Coruña, Spain, usc.es

## Abstract

Invasive fungal infections, especially those caused by *Candida* spp., have been classified as a serious global threat. The emergence of species intrinsically resistant to current drugs, along with the increase in acquired resistance, places significant pressure on the need to develop novel and more effective antifungal agents. A limited number of studies have shown the potential of palladium organometallic complexes as promising antifungal alternatives. Although the mechanism of antifungal activity of these complexes remains unaddressed, the findings support the idea that designing palladium (II) complexes could represent the next generation of antifungals. In this work, we synthesized four cyclopalladated complexes, **1a**, **1b**, **2a**, and **2b,** from Schiff base–amine phosphanes and evaluated their antifungal potential. Specifically, we assessed their spectrum of activity against several medically relevant *Candida* spp., their capacity to overcome resistance to current antifungal drugs, antibiofilm properties, uptake by fungal cells, in vivo toxicity, and intracellular effects. The most promising complexes, **1b** and **2b**, induce strong oxidative stress and lipid peroxidation, inhibit lipolysis, and disrupt vacuole integrity. Moreover, the rational design of the complexes allowed us to infer important structure–activity relationships. Our findings highlight the potential of palladium complexes as promising scaffolds for future antifungal therapeutic strategies and open new horizons for further development.

## 1. Introduction

Antimicrobial resistance (AMR), often referred to as the silent pandemic [1], is responsible for an unacceptable number of deaths each year [2]. If no actions are taken to address it, the annual death toll is estimated to reach 10 million by 2050 [3].

The management of AMR is particularly difficult in fungi, as the search for selective, nontoxic compounds is laborious and requires substantial financial investment [4]. This challenge arises from the close similarities between fungal and human cells compared with the differences between noneukaryotic microorganisms and human cells [5].

Invasive fungal infections caused by the yeast *Candida*, also known as invasive candidiasis, are associated with high rates of mortality and morbidity in hospitals [6]. The emergence of resistance among *Candida* spp. clinical isolates, along with the lack of therapeutic options, has contributed to this increasing incidence, leading the World Health Organization to include several *Candida* species in the critical group of its first‐ever fungal priority list and to urge the development of research into new antifungals [7, 8].

Although *Candida albicans* remains the most prevalent cause of invasive candidiasis, non–*C. albicans* species are emerging as serious clinical challenges [6]. *Candida parapsilosis* is frequently reported as the second most commonly isolated *Candida* spp. from blood cultures and poses a significant threat in neonatal intensive care units. This threat stems from its role as a typical commensal of human skin microbiota, with colonization of healthcare workers’ hands facilitating nosocomial transmission. Moreover, its ability to form biofilms on indwelling medical devices exacerbates the risk of invasive infections in this vulnerable population [9–11]. Another pressing issue is the emergence of the potent nosocomial and pan‐resistant pathogen *Candida auris*, which can colonize the skin and persist on the surfaces of medical devices, enabling person‐to‐person transmission and triggering outbreaks [12]. Several authors have suggested that its recent emergence might represent the first example of climate change selecting for thermotolerant yeasts with pathogenic potential [13–16].

Growing evidence suggests that metal complexes could be a promising alternative to tackle AMR [17–19]. They confer several advantages over organic compounds, specifically their unique physicochemical properties, such as variable oxidation states, redox activity, geometry, and ligand versatility, which allow rapid fine‐tuning of their design to develop structural variants with improved safety and antimicrobial activity [20].

**FIGURE 1:**
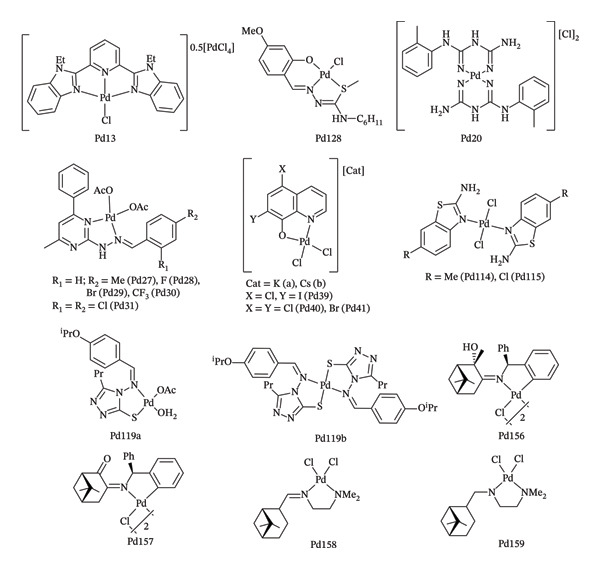
Representative examples of palladium complexes with antifungal activity.

Palladium, a rare element belonging to the platinum group of metals, has received attention in recent years due to its catalytic, mechanical, and electronic properties [21, 22]. In the medical field, palladium has been employed through complexes and nanoparticle conjugates, demonstrating anticancer and antibacterial activities [21, 23–26], while only a limited number of studies have explored the antifungal potential of palladium‐based organometallic complexes [27–34]. For instance, compounds incorporating tridentate pyridine, benzimidazole, biguanidine, hydrazone, or diazine‐based ligands have been reported (recently reviewed in [34]), and the structures of several representative examples are shown in Figure 1. Ionic palladium complexes incorporating tridentate pyridine–based ligands, such as Pd13 bearing a benzimidazole ligand, display high antifungal activity against *C*. *albicans* and *Cryptococcus neoformans* [35]. Salicylidene derivatives of thiosemicarbazones have also been used as polydentate ligands to generate chelate complexes, such as Pd128, which is active against *C. albicans* and *C. parapsilosis* [36]. In contrast, palladium complexes derived from aryl‐substituted biguanidine ligands (e.g., Pd20) show weaker activity against *C. albicans* [37]. Hydrazone‐based palladium complexes containing diazine cores (Pd28‐Pd31) exhibit a slightly superior antifungal activity than their corresponding initial ligand [38], whereas ionic diazine‐derived complexes (Pd39‐Pd41) show a level of activity similar to the initial ligands [39]. Several other palladium systems, including Pd114, Pd115, and Pd119a‐b, are weakly active [40, 41]. Palladium complexes Pd156–Pd159, including the bimetallic cyclopalladated complexes Pd156 and Pd157, have been reported to exhibit high antifungal activity against *C. albicans* and *C. neoformans* [42].

The chemistry of cyclopalladated complexes constitutes a part of organometallic chemistry that has attracted much interest in recent years due to their versatile structural and reactivity features as well as their properties and applications [43–47]. In the past, we have studied five‐membered cyclopalladated derivatives from bi‐, tri‐, and tetradentate ligands with nitrogen donor atoms. For example, Schiff bases react readily with Pd (II), Pt (II), and Pd(0) to give mono‐ and doubly cyclometallated complexes, and their reactivity with phosphanes has been extensively studied [48–52].

Tertiary polyphosphanes are essential ligands in coordination and in organometallic chemistry, owing to their wide range of steric and electronic properties. They are suitable for many applications, such as the development of new synthetic strategies, their use in medicine and pharmacy, or in the design of new products with interesting technological properties [53–56]. Thus, great interest has been directed toward the preparation of new polyphosphanes, which may be conveniently obtained by using multistage methods, but it may also be possible to develop new phosphane ligands through prior coordination of the phosphane precursor to an appropriate metal center. Typical examples of the latter are the C‐H acidity of the CH_2_ group in bis(diphenylphosphino)methane (dppm) [57–59] or the activated C=C group of coordinated 1,1‐bis(diphenylphosphino)ethene (vdpp). Uncoordinated vdpp is scarcely prone to nucleophilic attack and reacts directly with molecules, such as phosphanes and arsanes in the presence of a strong base or by quaternization of both phosphorus atoms [60, 61], but coordination to a metal fragment, e.g., M(CO)_4_ (M = Cr, Mo, W), MX_2_ (M = Pd, Pt; X = Me, AcO, Cl, I), or RuCl_2_ [62–64], increases the reactivity of the C=C bond toward the nucleophilic attack of HNu species.

We have previously reported the first addition products of cyclopalladated complexes via nucleophilic Michael addition to chelated vdpp, obtaining new phosphanes functionalized with 1,3‐dicarbonyl, amine, and alkoxy groups [65–67]. Herein, we widen the scope of our studies related to cyclometallated vdpp complexes, and we present their synthesis, full characterization, and antifungal properties.

## 2. Materials and Methods

### 2.1. General Information

Solvents were purified by standard methods [68]. We reported in previous papers the synthesis of the starting complexes [Pd{2,4‐(OMe)_2_C_6_H_2_C(H) = N(C_6_H_11_)C6,N}(μ‐Cl)]_2_ [66] and [Pd{3,4‐(OCH_2_O)C_6_H_2_C(H) N(C_6_H_11_)C2,N}(μ‐Cl)]_2_ [69]. Chemicals were used as supplied from commercial sources. Elemental analyses were carried out on a Carlo‐Erba elemental analyzer, Model 1108. IR spectra were recorded on a Thermo Scientific–Nicolet iS10. ^1^H and ^31^P‐{^1^H} NMR spectra were obtained as CDCl_3_ solutions and were recorded on a BRUKER WM‐250 and a JEOL‐FX‐100 spectrometers. All chemical shifts were reported downfield from standards.

### 2.2. Synthetic Procedures

Synthesis of [Pd{2,4‐(OMe)2C6H2C(H)=N(C6H11)C6,N}{(Ph2P)2C = CH2P,P}][PF6] (2). To a solution of [Pd{2,4‐(OMe)_2_C_6_H_2_C(H)=N(C_6_H_11_)C6,N}(μ‐Cl)]_2_ (162 mg, 0.208 mmol) in acetone (ca. 15 mL), vdpp (165 mg, 0.416 mmol) was added. The mixture was stirred for 2 h at room temperature, after which ammonium hexafluorophosphate (76 mg, 0.466 mmol) was added, the resultant solution stirred for a further 30 min, water (ca. 15 mL) added dropwise, and the resulting mixture stirred for 3 h. A pale yellow precipitate formed, which was filtered off, washed with water (ca. 5 mL), and dried in vacuo over anhydrous CaCl_2_. The desired complex was recrystallized from a dichloromethane/*n*‐hexane solution.

Yield: > 95%. Anal. Found: C, 54.9; H, 4.8; N, 1.5; C_41_H_42_F_6_NO_2_P_3_Pd requires C, 55.1; H, 4.7; N, 1.6. IR (*ν*_max_, cm^−1^): 1577 s (C=N). ^1^H NMR (250 MHz, CDCl_3_, δ in ppm, J in Hz): *δ* = 8.44 (d, 1H, Hi, ^4^J(PHi) = 8.2), 6.10 (d, 1H, H3, ^4^J(H3H5 = 2.0), 5.92 (m, 1H, H5, ^4^J(PH5) = 9.4, 7.3), 6.35 (m, 2H, CCH_2_), 3.80 (s, 3H, 2‐OMe), 3,24 (s, 3H, 4‐OMe). ^31^P‐{^1^H} NMR (250 MHz, CDCl_3_, δ in ppm, J in Hz) *δ* = 12.53 (d, P_trans−N_, ^2^J(PP) = 17.0), −4,29 (d, P_trans−c_), −147,91 (h, PF_6_^-^, J(PF) = 709,3).

Complex **1** was prepared similarly to complex **2** and isolated as a solid, but using [Pd{3,4‐(OCH_2_O)C_6_H_2_C(H)=N(C_6_H_11_)C2,N}(μ‐Cl)]_2_ and sodium perchlorate as appropriate.

[Pd{3,4‐(OCH2O)C6H2C(H)=N(C6H11)C2,N}{(Ph2P)2C=CH2P,P}][ClO4] (1). Yield: > 95%. Anal. Found: C, 57.9; H, 4.4; N, 1.5; C_40_H_38_ClNO_6_P_2_Pd requires C, 57.7; H, 4.6; N, 1.7. IR (*ν*_max_, cm^−1^): 1605 s (C=N). ^1^H NMR (250 MHz, CDCl_3_, δ in ppm, J in Hz): *δ* = 8.14 (d, 1H, Hi, ^4^J(PHi) = 8.1), 7.14 (dd, 1H, H6, ^3^J(H5H6) = 7.9, ^5^J(PH6) = 2.7), 6.58 (d, 1H, H5), 6,18 (m, 2H, CCH_2_), 5.07 (s, 2H, OCH_2_O). ^31^P‐{^1^H} NMR (250 MHz, CDCl_3_, δ in ppm, J in Hz) *δ* = 6.66 (d, P_trans−N_, ^2^J(PP) = 6.6), −4.05 (d, P_trans−C_).

[Pd{2,4‐(OMe)2C6H2C(H)=N(C6H11)C6,N}{(Ph2P)2CHCH2(4‐Me‐NC5H10)P,P}][PF6] (2a). To a solution of [Pd{2,4‐(OMe)_2_C_6_H_2_C(H)=N(C_6_H_11_)C6,N}{(Ph_2_P)_2_C=CH_2_P,P}][PF_6_] (**H**, 50 mg, 0.056 mmol) in dry benzene (ca. 10 mL), piperidine (8,1 μL, 0.068 mmol) was added. After the mixture was stirred for 6 h under argon, the solid formed was filtered off, washed with cold benzene, and dried in vacuo. The final product was isolated as pale orange needles by recrystallization from dichloromethane/*n*‐hexane.

Yield: 75.8%. Anal. Found: C, 57.2; H, 5.7; N, 2.6; C_47_H_55_F_6_N_2_O_2_P_3_Pd requires C, 56.8; H, 5.6; N, 2.8. IR (*ν*_max_, cm^−1^): 1576 m (C=N). ^1^H NMR (250 MHz, CDCl_3_, δ in ppm, J in Hz): *δ* = 8.43 (d, 1H, Hi, ^4^J(PHi) = 8.0), 6.04 (d, 1H, H3, ^4^J(H3H5 = 2.0), 5.67 (m, 1H, H5, ^4^J(PH5) = 10.2, 7.9), 4.64 (m, 1H,PCHP), 3.77 (s, 3H, 2‐OMe), 3.18 (s, 3H, 4‐OMe), 0.75 (d, 3H, 4‐Me, ^3^J(HH) = 6.5). ^31^P‐{^1^H} NMR (250 MHz, CDCl_3_, δ in ppm, J in Hz) *δ* = 10.19 (d, *P*_trans−N_, ^2^J(PP) = 54.9), −14.40 (d, *P*_trans−C_), −147,09 (h, PF_6_^-^, J(PF) = 709,0).

A similar method was used for the addition products **2b**, **1a**, and **1b**.

[Pd{2,4‐(OMe)2C6H2C(H)=N(C6H11)C6,N}{(Ph2P)2CHCH2(2‐Me‐NC5H10)P,P}][PF6] (2b). Yield: 77.4%. Anal. Found: C, 56.6; H, 5.3; N, 2.5; C_47_H_55_F_6_N_2_O_2_P_3_Pd requires C, 56.8; H, 5.6; N, 2.8. IR (*ν*_max_, cm^−1^): 1570 m (C=N). ^1^H NMR (250 MHz, CDCl_3_, δ in ppm, J in Hz): *δ* = 8.43 (d, 1H, Hi, ^4^J(PHi) = 8.1), 6.04 (d, 1H, H3, ^4^J(H3H5 = 2.0), 5.66 (m, 1H, H5, ^4^J(PH5) = 10.2, 7.9), 4.60 (m, 1H,PCHP), 3.77 (s, 3H, 2‐OMe), 3.17 (s, 3H, 4‐OMe), 1.19 (d, 3H, 2‐Me, ^3^J(HH) = 6.4). ^31^P‐{^1^H} NMR (250 MHz, CDCl_3_, δ in ppm, J in Hz) *δ* = 11.64 (d, *P*_trans−N_, ^2^J(PP) = 54.9), 9.67 (d, *P*_trans−N_, ^2^J(PP) = 54.9), −13.55 (d, *P*_trans−C_), −15.31 (d, *P*_trans−C_), −146,90 (h, PF_6_^-^, J(PF) = 709,1).

[Pd{3,4‐(OCH2O)C6H2C(H)=N(C6H11)C2,N}{(Ph2P)2CHCH2(4‐Me‐NC5H10)P,P}][ClO4] (1a). Yield: 81.7%. Anal. Found: C, 59.3; H, 5.6; N, 3.0; C_46_H_51_ClN_2_O_6_P_2_Pd requires C, 59.3; H, 5.5; N, 3.0. IR (*ν*_max_, cm^−1^): 1604 s (C=N). ^1^H NMR (250 MHz, CDCl_3_, δ in ppm, J in Hz): *δ* = 8.21 (d, 1H, Hi, ^4^J(PHi) = 8.1), 7.13 (dd, 1H, ^3^J(H5H6) = 7.7, ^5^J(PH6) = 2.5), 6.54 (d, 1H, H5), 5.06 (s, 2H, OCH_2_O), 4.49 (m, 1H,PCHP), 0.77 (d, 3H, 4‐Me, ^3^J(HH) = 6.4). ^31^P‐{^1^H} NMR (250 MHz, CDCl_3_, δ in ppm, J in Hz) *δ* = 0.27 (d, *P*_trans−N_, ^2^J(PP) = 51.4), −23.85 (d, *P*_trans−C_).

[Pd{3,4‐(OCH2O)C6H2C(H)=N(C6H11)C2,N}{(Ph2P)2CHCH2(2‐Me‐NC5H10)P,P}][ClO4] (1b). Yield: 75.7%. Anal. Found: C, 59.4; H, 5.7; N, 3.2; C_46_H_51_ClN_2_O_6_P_2_Pd requires C, 59.3; H, 5.5; N, 3.0. IR (*ν*_max_, cm^−1^): 1605 s (C=N). ^1^H NMR (250 MHz, CDCl_3_, δ in ppm, J in Hz): *δ* = 8.20 (d, 1H, Hi, ^4^J(PHi) = 8.2), 7.12 (dd, 1H, ^3^J(H5H6) = 7.8, ^5^J(PH6) = 2.5), 6.57 (d, 1H, H5), 5.06 (s, 2H, OCH_2_O), 4.45 (m, 1H,PCHP), 1.10 (d, 3H, 2‐Me, ^3^J(HH) = 6.2). ^31^P‐{^1^H} NMR (250 MHz, CDCl_3_, δ in ppm, J in Hz) *δ* = 3.24 (d, *P*_trans−N_, ^2^J(PP) = 63.5), 0.43 (d, *P*_trans−N_, ^2^J(PP) = 63.5), −16.19 (d, *P*_trans−C_), −23.04 (d, *P*_trans−C_).

### 2.3. Strains and Growth Conditions

The fungal strains used in this study are listed in Table 1. Strains were maintained on Sabouraud Dextrose Agar (SDA) plates. Unless otherwise indicated, all assays were performed in Roswell Park Memorial Institute (RPMI) 1640 medium with 0.165 M of MOPS buffer, adjusted to pH 7.0.

**TABLE 1:** Fungal strain used in this study.

Yeast strains	Source
*Candida albicans* SC5314	ATCC
*Candida glabrata* ATCC2001	ATCC
*Candida parapsilosis* ATCC22019	ATCC
*Candida dubliniensis* MYA 577	ATCC
*Candida tropicalis* ATCC7501	ATCC
*Candida krusei* ATCC6258	ATCC
*Candida auris* MYA 5001	ATCC
*Candida albicans* resistant to echinocandins (Case 3)	[70]
*Candida glabrata* resistant to fluconazole	Yeast strain evolved in laboratory

### 2.4. Antifungal Susceptibility Assays

Minimal inhibitory concentration (MIC) was determined by broth microdilution following the Clinical Laboratory and Standards Institute (CLSI) standard method M27‐A3 [71]. Serial dilutions of each Pd complex stock were prepared, with concentrations ranging from 50 to 0.05 μM. Growth in RPMI medium was recorded after 24 h at 35°C by measuring OD_530nm_ using the Epoch Microplate Spectrophotometer and BioTek Gen5 Data Analysis Software. Growth without complexity and with 1% DMSO (control condition) was used for normalization, after background subtraction (RPMI medium). The MIC_50_ was defined as the concentration at which the relative growth fell below 50% of the control condition. Assays were performed in biological triplicates. Minimal fungicidal concentration (MFC) was assessed by spotting 5 µL of the cultures onto YPD agar plates. Growth was recorded after 24 and 48 h of incubation at 35°C. The MFC was defined as the drug concentration that prevents yeast growth.

### 2.5. Measurement of Intracellular Palladium Levels

*Candida glabrata* ATCC2001 was grown to the exponential phase and left untreated or treated overnight with 20 μM of **1a**, **1b**, **2a**, and **2b**. Cells were harvested and washed with 10 mM EDTA and metal‐free water. Total intracellular palladium content was measured by inductively coupled plasma‐atomic emission spectroscopy (ICP‐AES) at REQUIMTE–LAQV, Universidade Nova de Lisboa, Caparica, Portugal. Data were normalized against OD_600nm._ All assays were performed in biological quadruplicates.

### 2.6. Biofilm Assays

The effect of the complexes on biofilms was assessed as previously described [72] with minor modifications. *C. albicans* SC5314 and *C. parapsilosis* ATCC7501 log phase cultures were diluted to 1 × 10^7^ cells/mL. Cells were added to each well of a black ibidi 24‐well plate with a #1.5H glass coverslip bottom and incubated at 37°C for 90 min to allow adherence. Nonadherent cells were aspirated, and the wells were washed with PBS. For the developmental inhibition assay, concentrations of the complexes prepared in RPMI corresponding to MIC, 10 μM and 20 μM, were added to the adherent cells and incubated at 37°C. After 24 h of incubation, confocal images were acquired using a Zeiss LSM 880 point scanning confocal microscope controlled by Zeiss Zen 2.3 (black edition) software, using a 10x EC Plan‐Neofluar 0.3 NA and a 63x Plan‐Apochromat 1.4NA DIC oil immersion objective (Zeiss). Image processing was performed using Fiji.

### 2.7. Reactive Oxygen Species (ROS) Measurements

Cells were grown to the exponential phase and left untreated or treated with 10 or 20 μM of **1b** or **2b**, or with 10 mM hydrogen peroxide for 2 h. Cultures (2 mL) were harvested, and the OD_600_ was normalized to the lowest measured value. Cells were then resuspended in PBS containing 5 μg/mL of dihydrorhodamine 123 (DHR123) and incubated at room temperature in the dark for 2 h. After incubation, cells were washed three times with PBS and transferred to a black NUNC microplate for fluorescence measurement.

### 2.8. Lipid Peroxidation Assay

Cells were grown to the exponential phase and left untreated or treated with 10 or 20 μM of **1b** or **2b**, or treated with 10 mM hydrogen peroxide for 1 h. After washing with PBS, glass beads were added, and samples were vortexed for 30 s and cooled on ice for 30 s. This process was repeated 10 times. Cell debris was removed by centrifugation at 4°C for 10 min, and the supernatant was collected and analyzed using the TBARS Assay Kit (Cayman Chemical, Ann Arbor, MI, USA) according to the manufacturer’s instructions.

### 2.9. Lipid Droplet Labeling

After treatment with the indicated concentration of the complexes, cultures were harvested by centrifugation, washed, and resuspended in PBS. For fluorescent labeling of lipid droplets, cells were incubated with BODIPY 493/503 at a final concentration of 5 µM for 15 min in the dark. After incubation, cells were washed three times and resuspended in PBS. Confocal Z‐series stacks were acquired using a Zeiss LSM 880 point scanning confocal microscope controlled by Zeiss Zen 2.3 (black edition) software, using a 63x Plan‐Apochromat 1.4NA DIC oil immersion objective (Zeiss). Image processing was performed using Fiji.

### 2.10. Lipolysis Inhibition

Cells were grown to the exponential phase and treated overnight with 20 μM of 1b or 2b or cadmium. Cell cultures were divided into two groups: In the first group (control group), cell cultures were harvested by centrifugation, washed, and resuspended in PBS with BODIPY 493/503 for fluorescent labeling of lipid droplets, as described above. In the second group, cells were treated with 50 μg/mL of cerulenin for 3 h. After incubation, cell cultures were harvested by centrifugation, washed, and resuspended in PBS with BODIPY 493/503 for fluorescent labeling of lipid droplets.

### 2.11. Vacuole Labeling

Cell cultures challenged with the indicated concentrations of the complexes were harvested by centrifugation, washed, and resuspended in PBS. Cells were then incubated with CMAC (7‐amino‐4‐chloromethylcoumarin) or FM4‐64 (N‐(3‐Triethylammoniumpropyl)‐4‐(6‐(4‐(Diethylamino) Phenyl) Hexatrienyl) Pyridinium Dibromide) at a final concentration of 100 and 40 µM, respectively, for 15 min in the dark. To confirm cellular viability, cells were also incubated with propidium iodide (PI) at a final concentration of 50 μg/mL, for 15 min. in the dark. After incubation, cells were washed and resuspended in PBS. Confocal Z‐series stacks were acquired using a Zeiss LSM 880 point scanning confocal microscope controlled by Zeiss Zen 2.3 (black edition) software, using a 63x Plan‐Apochromat 1.4NA DIC oil immersion objective (Zeiss). Image processing was performed using Fiji.

### 2.12. Zebrafish Husbandry and Care

Wild‐type zebrafish (*Danio rerio*) were housed at the animal facility at the University of Santiago de Compostela (REGA code ES270280346401) under standard aquaculture conditions: reverse osmosis water at 27°C (±1°C), pH 7.0 (±0.5), conductivity of 400–600 µS/cm, and a 14:10 h light–dark photoperiod. Breeding involved crossing an odd number of males with adult females, using marbles at the bottom of the tanks to enhance spawning conditions. Embryos were collected in Petri dishes with reverse osmosis water and examined under a Nikon TMS stereomicroscope to select viable specimens for toxicity assays.

### 2.13. Fish Embryo Acute Toxicity Test (FET)

Fertilized embryos obtained after natural spawning were maintained in Petri dishes until exposure to **1a**, **1b**, **2a**, and **2b**. The FET test was conducted following OECD Test Guideline 236 [73] with minor modifications. Embryos (*n* = 12 per replicate; three replicates per concentration) were exposed to test solutions of complexes **1a**, **1b**, **2a**, and **2b** prepared in embryo medium containing 1% DMSO. For complex **1a**, the concentrations tested were 0.005, 0.01, 0.1, 1, 10, 20, and 50 µM. For complex **1b**, the concentrations tested were 0.078, 0.150, 0.3125, 0.625, 1.25, 2.5, and 5 µM. For complex **2a**, the concentrations tested were 0.01, 0.1, 1, 10, 20, 50, and 100 µM. For complex **2b**, the concentrations tested were 1.5, 5, 10, 20, 40, 50, and 75 µM. Median lethal concentration (LC_50_) was determined 48 h postfertilization (hpf). All experiments were approved by the Animal Care and Use Committee of the University of Santiago de Compostela and conducted in accordance with Spanish regulations (CEEA‐LU‐003) and EU Directive 2010/63/EU. Mortality data were analyzed by probit analysis using ToxRat software (ToxRat Solutions GmbH, Alsdorf, Germany).

## 3. Results and Discussion

### 3.1. Structural Determination

**FIGURE 2:**
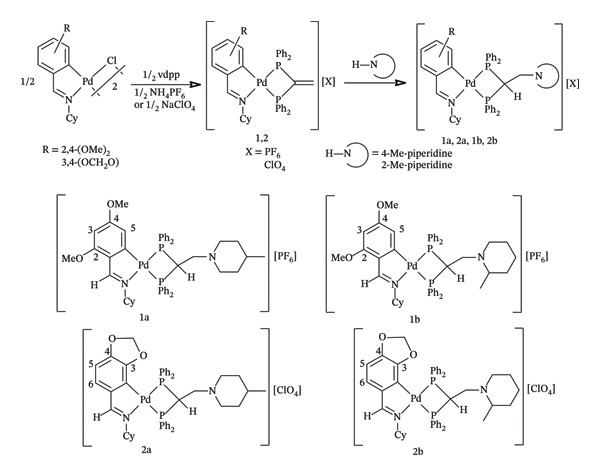
Reaction scheme for the synthesis of the Pd (II) complexes (up) and structure of the addition derivatives with numbering scheme (down).

Treatment of the chloro‐bridged cyclopalladated complexes [Pd{C_6_(R)H_2_C(H)=N(C_6_H_11_)}(μ‐Cl)]_2_ (R = 2,4‐(OMe)_2_ or 3,4‐(OCH_2_O)) with 1,1‐bis(diphenylphosphino)ethene and ammonium hexafluorophosphate or sodium perchlorate in a 1:2 M ratio in acetone yielded the mononuclear complexes [Pd{C_6_(R)H_2_C(H)=N(C_6_H_11_)}{(Ph_2_P)_2_C=CH_2_}][X] (X = PF_6_, **2**; ClO_4_, **1**), in which the diphosphane is acting as a bidentate chelate ligand bonding the palladium atom through the two phosphorus atoms. Reaction of **1** and 2 with the corresponding nucleophile in a 1:1 M ratio afforded the addition derivatives, which were isolated as air‐stable solids and were fully characterized (Figure 2).

The analytical data are consistent with the proposal structures. The spectroscopic data are in accordance with the maintenance of the cyclopalladated ring and the formation of a chelate ring with the diphosphane ligand. In complexes **2** and **1**, as well as in their addition derivatives, the IR spectra showed bands at 850 br and 1080 s cm^−1^, arising from the corresponding counterions, PF_6_^−^ and ClO_4_^−^, respectively. Nitrogen coordination to the metal center was supported by the shift toward lower frequency in the IR spectra of the *ν*(C=N) stretching vibration with respect to the corresponding one in the free imines [74].

The HC=N resonance in the ^1^H NMR spectra appeared as a doublet due to coupling with the ^31^P nucleus trans to nitrogen (^4^J(PH) ca. 8.0 Hz) and upfield shifted from its position in the spectra of the free ligands [75]. In complexes **2**, **2a**, and **2b**, the H5 resonance appeared as a multiplet by coupling to H3 and to both ^31^P nuclei (^4^J(*P*_trans−N_H) > ^4^J(*P*_trans−C_H)). In complexes **1**, **1a**, and **1b,** the signal assigned to the hydrogen atom in *ortho* to the imine group nucleus (H6) appeared as a doublet by coupling to only the phosphorus atom *trans* to carbon (^5^J(*P*_trans−C_H) ca. 2.5 Hz). The H5 and 4‐OMe (“1” derivatives) and OCH_2_O (“2” derivatives) proton signals were shifted to lower frequency by ca. 0.7 ppm, with respect to their position in the spectra of the starting products, due to the shielding effect of the phosphane phenyl rings cis to the metallated carbon atom, as we have found in related systems [66, 76, 77].

The ^31^P‐{^1^H} NMR spectra of the mononuclear complexes showed two doublets, indicating the two phosphorus atoms to be nonequivalent in each case, and also showed the resonance due to the phosphorus nucleus in the hexafluorophosphate ion as a heptuplet ca. −147 ppm (^1^J(PF) ca. 710 Hz). The assignment of the doublets was made in accordance with the assumption that a ligand of greater trans influence shifts the resonance of the phosphorus atoms *trans* to it to lower frequency [78, 79] and was confirmed by selective decoupling experiments. In the case of the addition complexes **2b** and **1b**, the 2‐methylpiperidine derivatives, the ^31^P‐{^1^H} NMR spectrum showed two pairs of doublets, in accordance with the existence of optical isomers due to the presence of two stereogenic centers: the C_α_ atom with respect to both phosphorus atoms and the C‐Me atom of the piperidine ring. We observed this behavior in addition reactions with asymmetric nucleophiles, such as 1,3‐dicarbonyl compounds [66, 80].

The comparison of the NMR spectra allows to confirm unambiguously the addition reaction: (i) The resonance of the vinylidene protons (*δ*(C=CH_2_) ca. 6.2 ppm) disappeared in the ^1^H‐NMR spectra of the addition derivatives, and a multiplet ca. 4.5 ppm was assigned to the P_2_CH proton; (ii) the coupling constant between the phosphorus atoms in the ^31^P‐{^1^H} NMR spectra of the addition derivatives, ^2^J(PP) = 51–64 Hz, is greater than the one observed for the starting complexes, ^2^J(PP) = 17.0 and 6.6 Hz for **2** and **1**, respectively. A similar behavior was observed in other cyclopalladated complexes, and we believe this may be due to the greater contribution of ^2^J(PP) across the metal center (normally of negative sign) in the addition products than in **2** and **1**, thus lowering the absolute value of the coupling constant. The ^31^P resonances of the coordinated diphosphane ligand are upfield shifted according to the expected influence of the four‐membered ring, which forms in the coordination sphere of the palladium atom [81]. Thus, the withdrawing effect of the cyclopalladated moiety derived from a Schiff base ligand suffices to obtain the addition product due to the induced polarization of the C=CH_2_ double bond in the starting complexes and to the relief of angle strain at the carbon adjacent to both phosphorus atoms after addition.

### 3.2. The Newly Synthesized Cyclopallated Complexes Show Promising Antifungal Activity

The susceptibility of medically relevant *Candida* spp. to the four palladium complexes (**1a**, **1b**, **2a**, and **2b**) was analyzed by determining their MICs according to CLSI guidelines [71]. Of the four complexes, **1a** was generally the least active, with MIC values ranging from 6.25 to 25 μM (Table 2). The remaining complexes showed stronger antifungal activity, with MIC values spanning 0.78 to 6.25 μM. Among the tested species, *Candida dubliniensis* was the most susceptible to the complexes. Within each family, diphosphane functionalization with 2‐methyl‐piperidine (**1b** and **2b**) enhances antifungal activity compared to 4‐methyl‐piperidine (**1a** and **2a**), with this effect being more pronounced in family **1**. Of note, while our results indicate that amine functionalization enhances activity, it would be interesting to evaluate the antifungal behavior of unfunctionalized cyclopalladated complexes (i.e., without piperidine addition). Interestingly, all complexes were capable of overcoming resistance to fluconazole (a triazole) and caspofungin (an echinocandin), two widely used antifungal drugs [82], in *C. glabrata* and *C. albicans*, respectively. They also overcame the intrinsic resistance of *C. glabrata* and *Candida krusei* to fluconazole (Table 2).

**TABLE 2:** Minimal inhibitory concentration (MIC) values of the complexes against medically relevant *Candida* spp.

Yeast strain	MIC (μM)				
	1a	1b	2a	2b	Fluconazole
*Candida albicans*	12.5	3.13	6.25	3.13	1.63
*Candida glabrata*	25	3.13	6.25	3.13	6.52
*Candida parapsilosis*	25	3.13	3.13	1.56	3.27
*Candida dubliniensis*	6.25	0.78	0.78	1.56	0.82
*Candida krusei*	12.5	1.56	3.13	1.56	52.24
*Candida tropicalis*	12.5	3.13	3.13	1.56	1.63
*Candida auris*	25	3.13	3.13	1.56	3.27
*Candida albicans* resistant to echinocandins^∗^	25	3.13	6.25	3.13	0.41
*Candida glabrata* resistant to fluconazole	25	3.13	6.25	3.13	> 417.9

^∗^Minimal inhibitory concentration for caspofungin is 3.3 μM.

**FIGURE 3:**
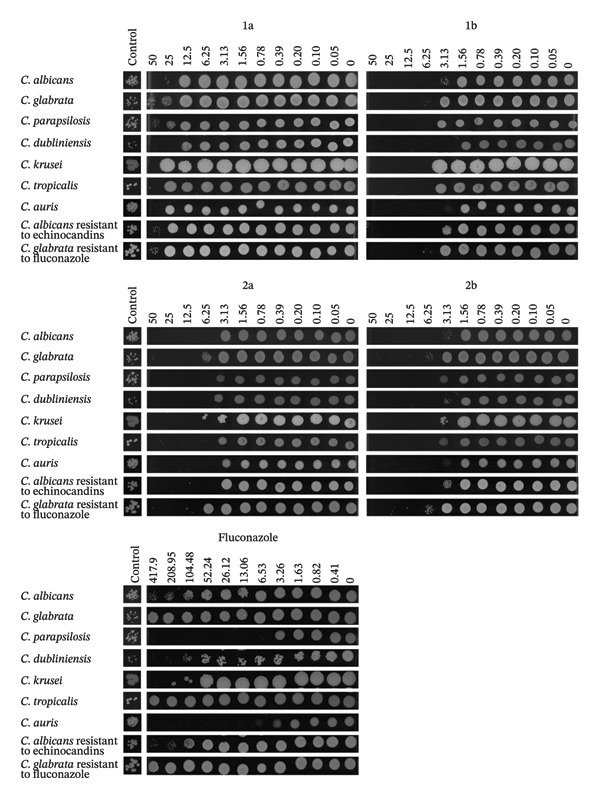
Complexes 1a, 1b, 2a, and 2b are fungicidal at concentrations close to their MIC. *Candida* cultures were incubated with the specified concentrations (μM) of 1a, 1b, 2a, 2b, or fluconazole for 24 h, and 5 μL of each culture was spotted onto YPD agar plates. Images were recorded after 24 h of incubation. Control—initial inoculum before compound addition.

To assess whether the antifungal activity of the palladium complexes was fungistatic or fungicidal, cultures of each *Candida* spp. were exposed to several concentrations of the complexes, and 5 μL was spotted onto YPD agar plates to determine the MFC (Figure 3). After 24 h at 30°C, growth in each spot was compared to that of the initial inoculum (control, Figure 3), and the MFC was defined as the lowest drug concentration that resulted in reduced growth relative to this control. Consistent with the MIC results (Table 2), **1a** displayed the highest MFCs across all species tested. In contrast, **1b**, **2a**, and **2b** showed MFCs only onefold to twofold higher than their MICs, indicating potent fungicidal activity, superior to that of the widely used antifungal fluconazole, for which fungicidal activity against *C. albicans*, *C. glabrata*, *C. dubliniensis*, and *Candida tropicalis* was only observed at concentrations 64‐fold higher than the MIC.

### 3.3. The Cyclopalladated Complexes Accumulate Within Yeast Cells

**FIGURE 4:**
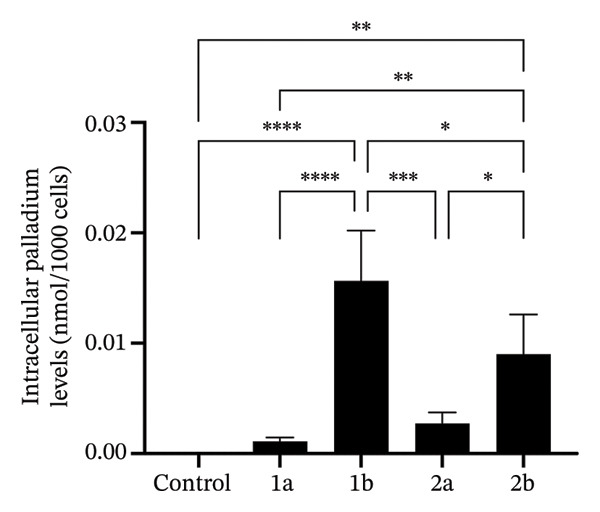
Complexes 1b and 2b accumulate at higher levels than 1a and 2a in *C. glabrata*. The palladium content of *C. glabrata* cells, either left untreated (control) or treated with 20 μM of each complex, was measured by ICP‐AES. Results represent the mean of biological quadruplicates ± standard deviation. Statistical significance was assessed using one‐way ANOVA with Turkey’s HSD post hoc test (^∗^ *p* < 0.05, ^∗∗^ *p* < 0.01, ^∗∗∗^ *p* < 0.001, ^∗∗∗∗^ *p* < 0.0001).

To investigate whether the complexes accumulated inside the yeast cells, we performed ICP‐AES to quantify the intracellular levels of palladium in *C. glabrata* after treatment with **1a**, **1b**, **2a**, and **2 b**. *C. glabrata* was selected for these experiments because its lack of hyphae formation prevents cell aggregation, which allows more accurate OD_600_‐based normalization in RPMI medium. As metal dissociation from the complexes is unlikely, this method provides an indirect assessment of complex internalization and accumulation. Cells were treated with the same concentration of each complex (20 μM) to facilitate the correlation between results. All four palladium complexes are able to enter the yeast cells, but their accumulation levels differ, with compounds **1b** and **2b** showing the highest accumulation (Figure 4).

Complexes containing 2‐methylpiperidine (**1b** and **2b**) were generally more active than those containing 4‐methylpiperidine (**1a** and **2a**) (Table 2). This correlates with the lower intracellular accumulation observed for the latter (Figure 4). The 2‐methylpiperidine group increases lipophilicity more than the 4‐methyl analog, potentially improving membrane permeability. **1a** was the least active complex, which is consistent with its lower intracellular accumulation (Figure 4).

Based on these findings, complexes **1b** and **2b** were selected for further studies.

### 3.4. 1b and 2b Have Antibiofilm Activity and Inhibit Filamentation

One major virulence factor of *Candida* spp. is the formation of biofilms on host cells and abiotic surfaces [83]. These structures, which are intrinsically more resistant to antifungals, impede the effective treatment of the infection [83]. Gayatri et al. synthesized thiazolinyl‐picolinamide–based palladium (II) complexes that were active against *C. albicans* and *C*. *auris*, and demonstrated their ability to inhibit biofilm formation in both species [30]. Here, we extend this observation to *C. parapsilosis*, whose biofilm formation poses a serious threat to neonates [10].

**FIGURE 5:**
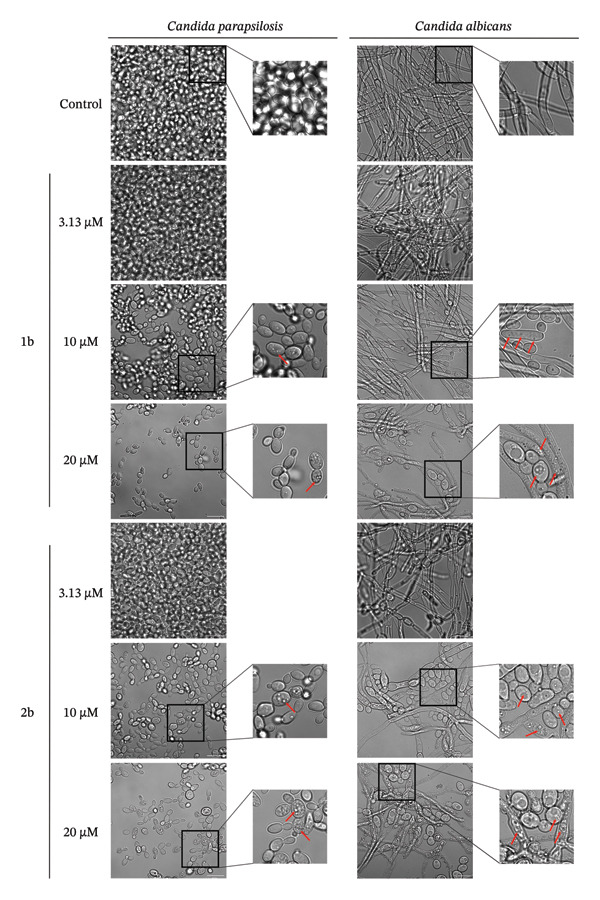
Palladium complexes exhibit antibiofilm activity. Confocal microscopy images of *C. parapsilosis* and *C. albicans* biofilms, either untreated (control) or treated with the indicated concentrations of 1b and 2b. The scale bar represents 10 μm.

We observed a significant antibiofilm effect of **1b** and **2b** on *C. albicans* and *C. parapsilosis*, with **2b** showing a more pronounced effect at lower concentrations (Figure 5). Notably, both complexes affected *C. albicans* filamentation, with concentrations of 10 μM or higher causing a marked reduction in filamentation. This is a significant finding, as filamentous yeast forms are crucial not only for biofilm development but also for infection dissemination [84]. It is tempting to speculate that, at least in *C. albicans*, these complexes might interfere with the expression of genes involved in phenotypic switching from yeast to hyphae.

### 3.5. Treatment With 1b and 2b Induces the Formation of Reactive Oxygen Species and Lipid Droplets

A careful inspection of the microscopy images in Figure 5 revealed the accumulation of small rounded structures following treatment with **1b** and **2b**, which we confirmed to be lipid droplets using BODIPY 493/503 and confocal microscopy (Figures 6(a) and 6(b)). In the absence of the complexes (control, Figures 6(a) and 6(b)), BODIPY 493/503 predominantly localized to a single lipid droplet under control conditions, whereas treatment with **1b** or **2b** led to its redistribution into numerous similar structures.

FIGURE 61b and 2b induce reactive oxygen species and lipid peroxidation. (a) *C. parapsilosis* and (b) *C. albicans* cells, either untreated or treated with 1b and 2b, were stained with BODIPY 493/503 to visualize lipid droplets by confocal microscopy. The scale bar represents 5 μm. (c) Intracellular ROS levels in *C. parapsilosis* cells, untreated (control) or treated with 1b or 2b, were quantified using the fluorescent dye dihydrorhodamine 123 (DHR 123). (d) Lipid peroxidation was assessed by measuring malondialdehyde (MDA) levels in *C. parapsilosis* cells, untreated (control) or treated with 1b or 2b. Results represent the mean of four replicates ± standard deviation. Statistical significance was assessed using one‐way ANOVA with Turkey’s HSD post hoc test (^∗^ *p* < 0.05, ^∗∗^ *p* < 0.01, ^∗∗∗^ *p* < 0.001, ^∗∗∗∗^ *p* < 0.0001).

**Figure:**
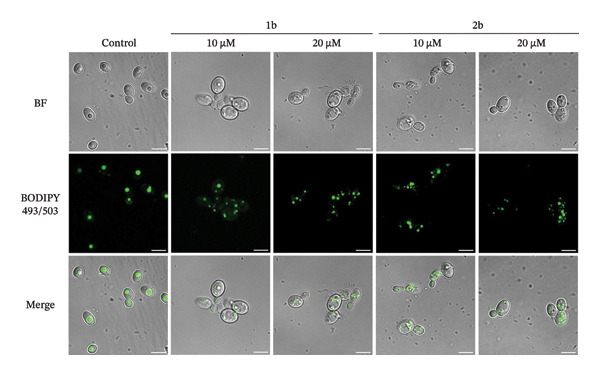
(a)

**Figure:**
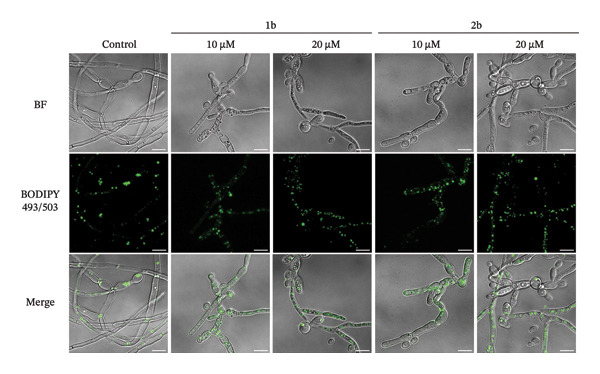
(b)

**Figure:**
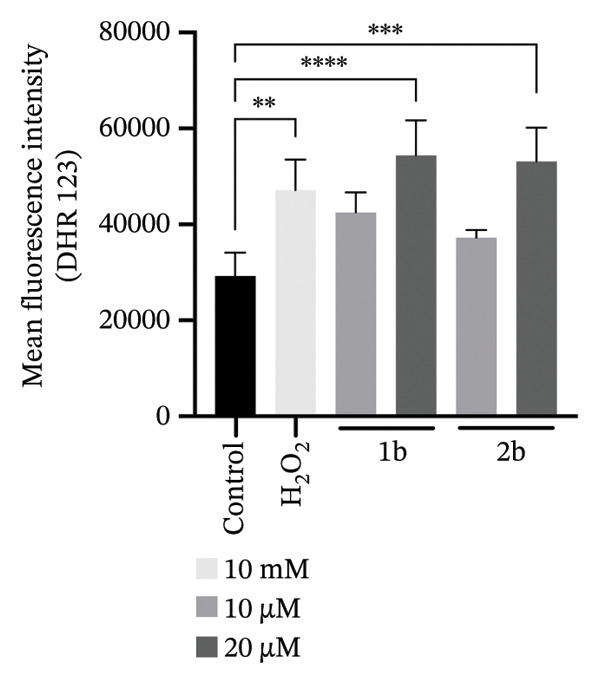
(c)

**Figure:**
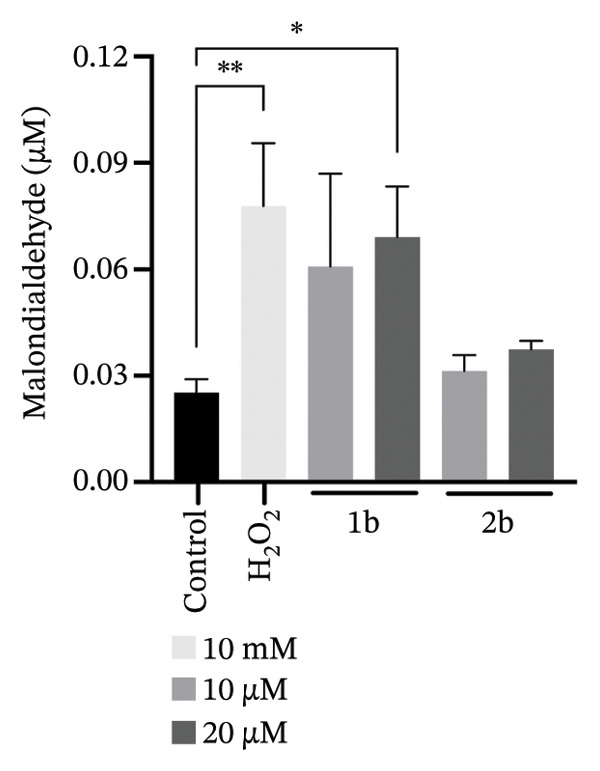
(d)

Lipid droplets are organelles derived from the endoplasmic reticulum, specialized in the regulation of lipid homeostasis, serving as a source of membrane components and energy, and protecting cells from lipotoxicity [85, 86]. In mammalian cells, it is thought that lipid droplets are induced not only to maintain endoplasmic reticulum homeostasis or during starvation, but also in response to oxidative stress. Although the mechanism remains to be elucidated, during this stress, phospholipases may release polyunsaturated fatty acids (PUFAs) from membranes to be stored in lipid droplets, where they are less susceptible to lipid peroxidation [87]. In yeast cells, membrane stress triggers the release of free fatty acids, which can be saturated or unsaturated fatty acids (UFAs) [88, 89].

We therefore asked whether the observed accumulation in lipid droplets represents a cellular response to increased ROS and consequent increase in lipid peroxidation. Indeed, palladium complexes are known to generate ROS in mammalian cells [90, 91], and palladium nanoparticles have been shown to trigger ROS production in *C*. *albicans* [24]. To test whether this also holds true for *C. parapsilosis*, cells were treated with **1b** and **2b** and ROS levels were measured using dihydrorhodamine 123 (DHR123), and lipid peroxidation was assessed via the thiobarbituric acid reactive substance (TBARS) assay to measure malondialdehyde (MDA), a reactive aldehyde formed as a byproduct of lipid peroxidation (Figures 6(c) and 6(d), respectively) [92]. **1b** and **2b** increased ROS levels and lipid peroxidation; in the case of **1b**, the effect was similar to that induced by a 1000‐fold higher concentration of hydrogen peroxide. Therefore, the increased number of lipid droplets observed upon treatment may result from the release of UFAs into these organelles as a mechanism of protection. An analogous effect was reported by Nachiappan and colleagues [93] under cadmium‐induced oxidative stress in *Saccharomyces cerevisiae* and was also observed by us in this study (Figure 6). Similar results with palladium complexes were documented by Fahmy and colleagues [29] in HeLa cells. Remarkably, the authors showed that the type of metal and the nature of the ligand influenced the MDA levels, with a copper complex producing a more pronounced effect [29]. These findings could guide the further development of our complexes.

**FIGURE 7:**
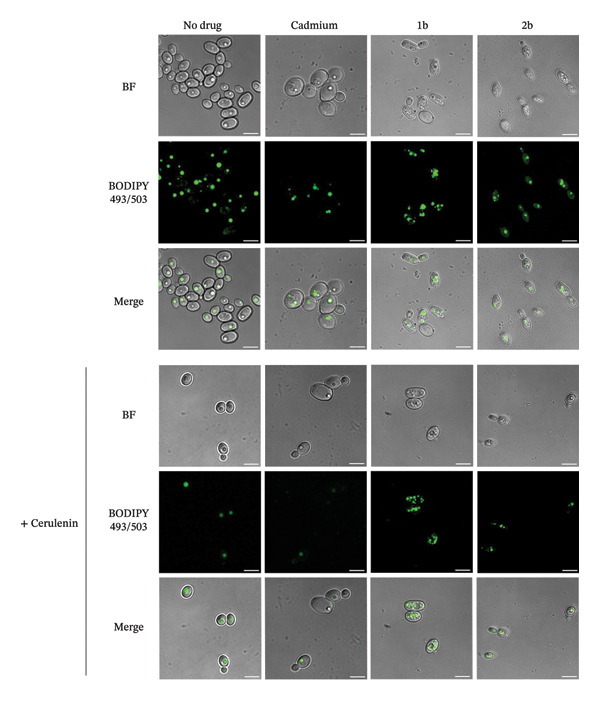
Lipolysis is inhibited by 1b and 2 b. *C. parapsilosis* cells, either untreated or treated with 20 μM of cadmium, 1b or 2b, were stained with BODIPY 493/503 to compare lipid droplet accumulation before and after a 3‐h treatment with 50 μg/mL of cerulenin using confocal microscopy. The scale bar represents 5 μm.

While the accumulation of lipid droplets induced by **1b** can be attributed to increased lipid peroxidation (Figure 6(d)), this explanation does not fully account for the effects of **2b**, as MDA levels did not differ greatly from the control (Figure 6(d)), despite elevated ROS levels (Figure 6(b)). Thus, we investigated whether **1b** and **2b** could also impair lipolysis, i.e., the cellular reutilization of lipid droplets [85]. Given that vacuoles play a central role in lipolysis [85], we also evaluated vacuolar morphology following treatment with **1b** and **2b**. For this experiment, cell cultures previously treated with **1b** and **2b** were subsequently exposed to cerulenin, a specific inhibitor of fatty acid and sterol synthesis [94], to force cells to utilize their lipid droplets. Cadmium was used as a positive control (Figure 7). In the presence of cadmium, lipid droplets formed, but their levels decreased following cerulenin treatment, indicating that cells could reutilize them to restore fatty acid levels. In contrast, treatment with **1b** and **2b** resulted in persistent lipid droplet accumulation, even after cerulenin exposure (Figure 7).

**FIGURE 8:**
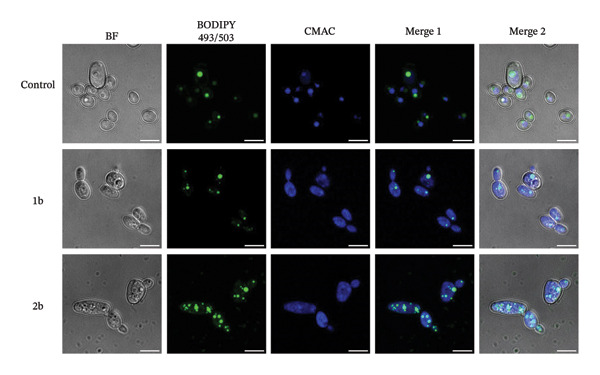
In *C. parapsilosis,* vacuole integrity is compromised after treatment with 1b and 2 b. *C. parapsilosis* cells left untreated or treated overnight with 10 μM of 1b or 2b were stained with BODIPY 493/503 and CMAC to visualize lipid droplets and vacuoles, respectively, by confocal microscopy. The scale bar represents 5 μm.

Furthermore, CMAC and FM4‐64 staining revealed that vacuoles became disrupted following **1b** and **2b** treatment (Figure 8 and Figure S1). Importantly, we verified that the loss of vacuole integrity was not caused by cell death induced by the compounds (Figure S2). Although the underlying molecular mechanism remains to be explored, the disruption of vacuolar integrity may precede the accumulation of lipid droplets and contribute to the toxicity of the complexes, as excess lipid droplets are normally cleared via vacuoles [85].

These results suggest that **1b** and **2b** increase cellular ROS and promote lipid droplet formation by enhancing lipid peroxidation and/or inhibiting lipolysis through the disruption of yeast vacuoles.

### 3.6. In Vivo, 2b Exhibits Lower Toxicity

The zebrafish (*Danio rerio*) has become an established and powerful model organism in numerous biomedical disciplines, particularly in toxicology [95]. The FET test (OECD 236) [73] was employed to evaluate the toxicological profile of the four complexes. Median lethal concentrations (LC_50_) at 48 h postfertilization (hpf) were determined following standard procedures (Figure 9).

FIGURE 9In vivo toxicity. The fish embryo acute toxicity (FET) test (OECD 236) was employed to evaluate the toxicological profile of 1b (a), 2b (b), 1a (c), and 2a (d).

**Figure:**
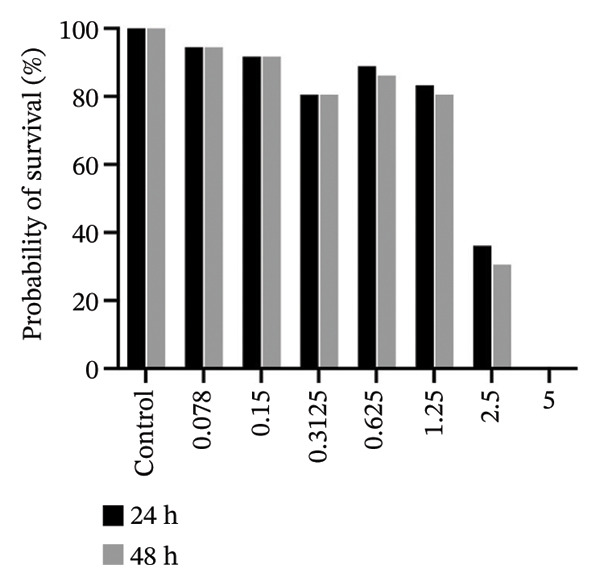
(a)

**Figure:**
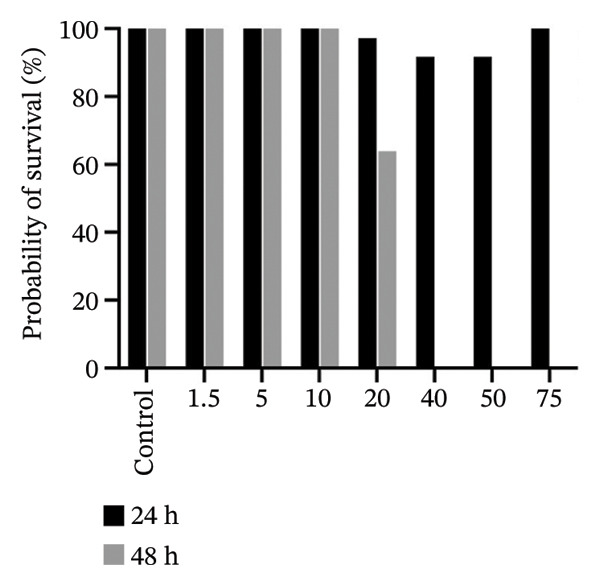
(b)

**Figure:**
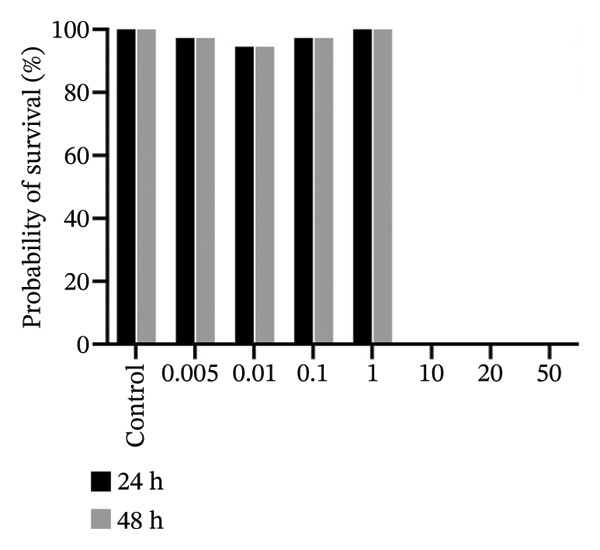
(c)

**Figure:**
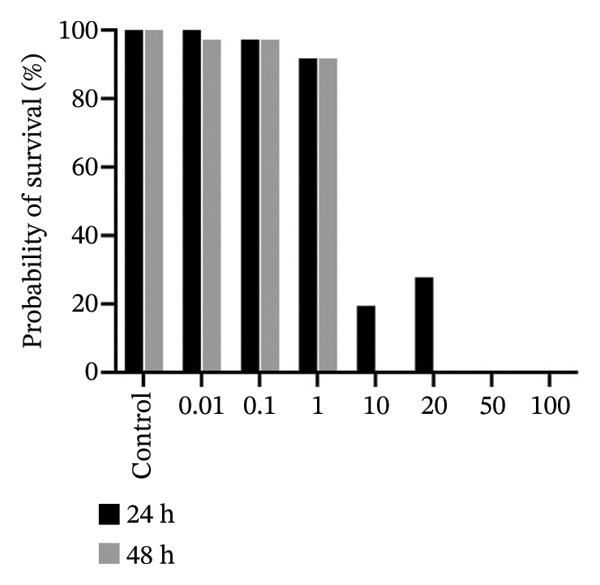
(d)

The results reveal a clear difference in acute toxicity. **1a**, **1b**, and **2a** exhibited high toxicity, with an LC_50_ of 1.36, 1.75, and 1.78 µM, respectively, indicating potent lethal effects at low micromolar concentrations. In contrast, **2b** displayed lower toxicity, with an LC_50_ of 20.98 µM, representing more than a 10‐fold difference in toxicity compared to the other complexes (Figure 9). These findings suggest that the imine moiety used to functionalize the disphosphane strongly affected in vivo toxicity, with 4‐methylpiperidine (**1a** and **2a**) enhancing toxicity in comparison with those containing 2‐methylpiperidine (**1b** and **2b**) (Figure 9). Binh et. al., which synthesized artemisinin triazole derivatives, observed a higher IC_50_ with a compound possessing 4‐methylpiperidine against four cell lines in comparison with the compound with 2‐methylpiperidine [96]. Interestingly, an opposite pattern of toxicity was observed for **2b** in yeast cells, with **2b** being more toxic (transducing in lower MICs) (Table 2). These differences suggest distinct mechanisms of action and/or differential uptake by different eukaryotic cells and could be further exploited to harness the antifungal potential of cyclopalladated complexes in future studies.

## 4. Conclusions

The four Schiff base–derived cyclopalladated bearing amine‐functionalized phosphane complexes described here were active against multiple *Candida* spp. and, importantly, exhibited fungicidal activity, a feature particularly noteworthy, as invasive candidiasis is an opportunistic infection that greatly affects immunocompromised patients [6], who cannot rely on their immune system to fight the infection.

Overall, the data presented here highlight palladium complexes as promising antifungal strategies capable of overcoming resistance to current agents. Structure–activity relationship studies and toxicity assays place **2b** in the spotlight as a promising scaffold for the future development of this class of organometallic complexes.

## Funding

This work was supported by the FCT–Foundation for Science and Technology, I.P., through MOSTMICRO‐ITQB R&D Unit (doi.org/10.54499/UID/04612/2025, UIDP/PRR/04612/2025), and LS4FUTURE Associated Laboratory (doi.org/10.54499/LA/P/0087/2020). O.L‐R acknowledges FCT, POPH—Programa Operacional Potential Humano, and FSE (European Social Fund) for the CEEC 2017 Initiative (https://doi.org/10.54499/CEECIND/04566/2017/CP1428/CT0009). CM‐L was recipient of a PhD fellowship supported by the FCT, with reference UI/BD/153387/2022 (https://doi.org/10.54499/UI/BD/153387/2022). The work was also partially supported by the PPBI—Portuguese Platform of BioImaging (PPBI‐POCI‐01‐0145‐FEDER‐022122), cofunded by national funds from OE—“Orçamento de Estado” and by European funds from FEDER.

## Conflicts of Interest

The authors declare no conflicts of interest.

## Supporting Information

Fig. S1 In C. parapsilosis, vacuole integrity is compromised after treatment with 1b and 2 b. C. parapsilosis cells left untreated or treated overnight with 10 μM of 1b or 2b were stained with BODIPY 493/503 and FM4‐64 to visualize lipid droplets and vacuoles, respectively, by confocal microscopy. The scalebar represents 5 μm.

Fig. S2 The loss of vacuole integrity does not correlate with cell death. Confocal microscopy images of *Candida parapsilosis* cells either left untreated or treated with 10 μM of compounds 1b or 2b. Cells were stained with CMAC to visualize vacuoles and Propidium Iodide (PI) to indicate membrane‐compromised (dead) cells. The scalebar represents 5 μm.

## Supporting information


**Supporting Information** Additional supporting information can be found online in the Supporting Information section.

## Data Availability

All data supporting the findings of this study are available within the paper and its Supplementary Information. All other data are available from the corresponding author upon request.
