# Supplementary material for: Cyclopalladated Complexes With Functionalized Diphosphanes as Promising Antifungal Scaffolds
Source: Bioinorg Chem Appl. 2026 Apr 11;2026:6220526. doi: 10.1155/bca/6220526 (PMC13069970; doi:10.1155/bca/6220526)
Supplement: Supplementary file 1 — Supporting Information Additional supporting information can be found online in the Supporting Information section. [file BCA-2026-6220526-s001.pdf]

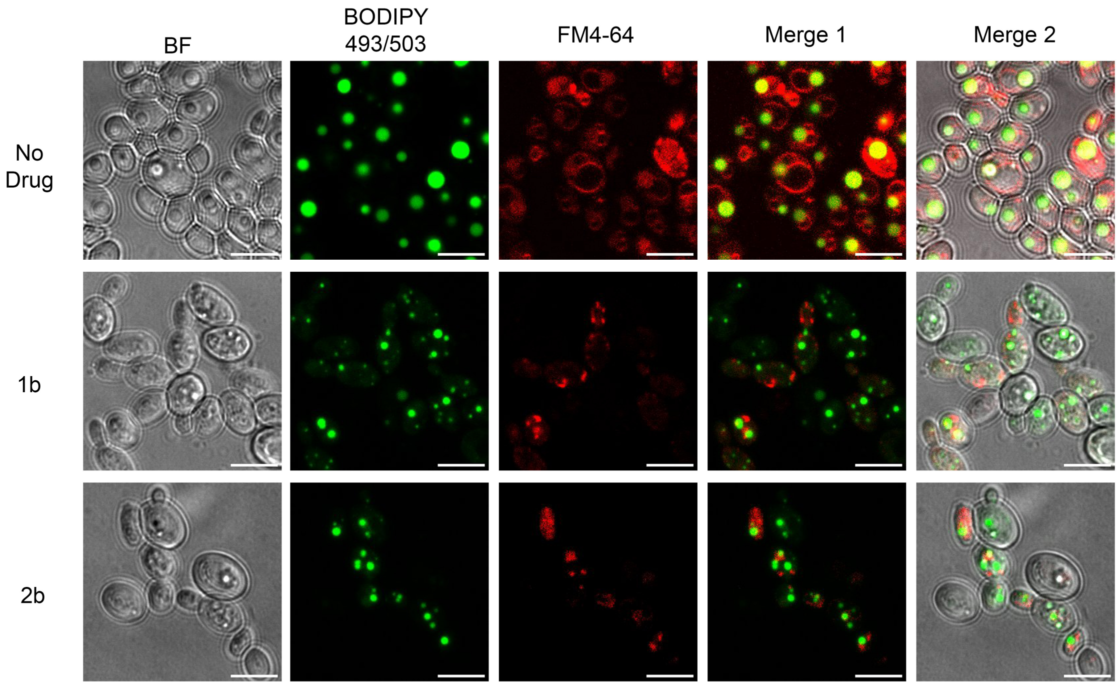

Fig. S1 In *C. parapsilosis*, vacuole integrity is compromised after treatment with 1b and 2b. *C. parapsilosis* cells left untreated or treated overnight with 10 µM of 1b or 2b were stained with BODIPY 493/503 and FM4-64 to visualize lipid droplets and vacuoles, respectively, by confocal microscopy. The scalebar represents 5 µm.

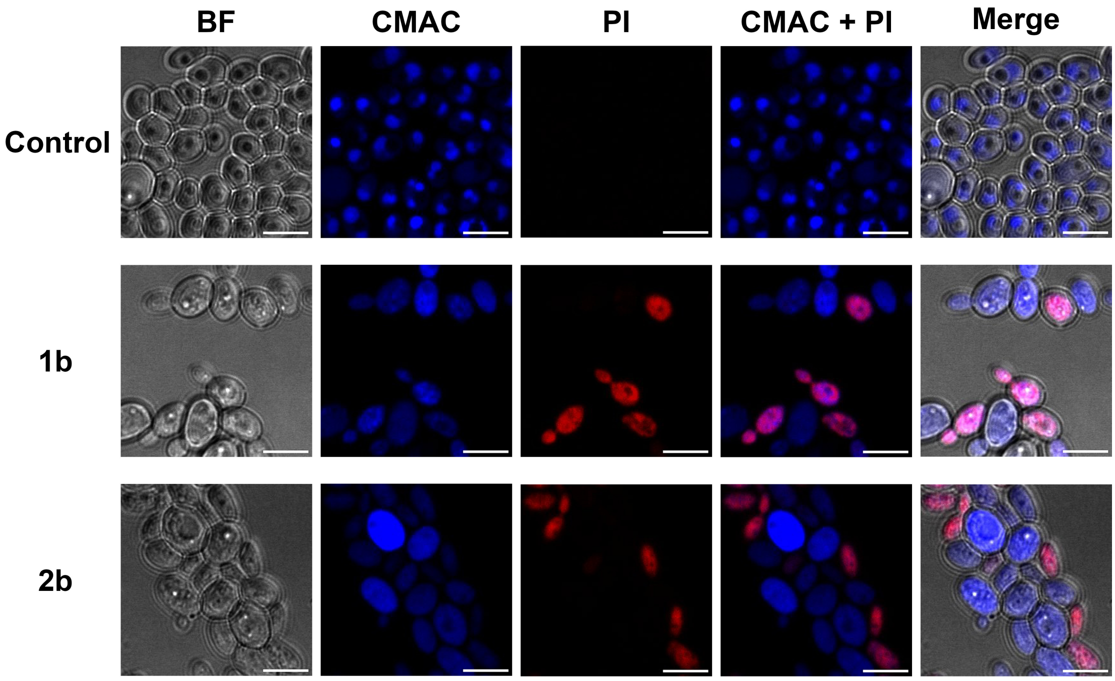

Fig. S2 The loss of vacuole integrity does not correlate with cell death. Confocal microscopy images of *Candida parapsilosis* cells either left untreated or treated with 10 µM of compounds 1b or 2b. Cells were stained with CMAC to visualize vacuoles and Propidium Iodide (PI) to indicate membrane-compromised (dead) cells. The scalebar represents 5 µm.
